# Sustainability of breastfeeding interventions to reduce child mortality rates in low, middle-income countries: A systematic review of randomized controlled trials

**DOI:** 10.3389/frhs.2022.889390

**Published:** 2022-08-11

**Authors:** Alexis Engelhart, Stacey Mason, Ucheoma Nwaozuru, Chisom Obiezu-Umeh, Victoria Carter, Thembekile Shato, Titilola Gbaja-Biamila, David Oladele, Juliet Iwelunmor

**Affiliations:** ^1^Department of Behavioral Science and Health Education, College for Public Health and Social Justice, Saint Louis University, Saint Louis, MO, United States; ^2^Department of Implementation Science, Wake Forest School of Medicine, Winston-Salem, NC, United States; ^3^Department of Social Work, College for Public Health and Social Justice, Saint Louis University, Saint Louis, MO, United States; ^4^Implementation Science Center for Cancer Control and Prevention Research Center, Brown School, Washington University in St. Louis, Saint Louis, MO, United States; ^5^Department of Surgery (Division of Public Health Sciences), Washington University School of Medicine, Washington University in St. Louis, Saint Louis, MO, United States; ^6^Clinical Sciences Division, Nigerian Institute of Medical Research, Lagos, Nigeria

**Keywords:** sustainability, breastfeeding interventions, child mortality, low- and middle-income countries, randomized controlled trial

## Abstract

Child mortality is the lowest it has ever been, but the burden of death in low- and middle-income countries (LMICs) is still prevalent, and the numbers average above the global mean. Breastfeeding contributes to the reduction of child mortality by improving chance of survival beyond childhood. Therefore, it is essential to examine how evidence-based breastfeeding interventions are being maintained in resource-constrained settings. Guided by Scheirer and Dearing's sustainability framework, the aim of this systematic review was to explore how evidence-based breastfeeding interventions implemented to address child mortality in LMICs are sustained. The literature search included randomized controlled trials (RCTs) of breastfeeding interventions from the following electronic databases: Cochrane Library, Global Health, PubMed, Scopus, and Web of Science. Literature selection and data extraction were completed according to the PRISMA guidelines. A narrative synthesis was used to investigate factors that contributed to sustainability failure or success. A total of 497 articles were identified through the database search. Only three papers were included in the review after the removal of duplicates and assessment for eligibility. The three RCTs included breastfeeding interventions predominately focusing on breastfeeding initiation and exclusivity in rural, semi-rural, and peri-urban areas in South Africa, Kenya, and India. The number of women included in the studies ranged from 901 to 3,890, and the duration of studies stretched from 6 weeks to 2.5 years. In two studies, sustainability was reported as the continuation of the intervention, and the other study outlined program dissemination and scale-up. Facilitators and barriers that influenced the sustainability of breastfeeding interventions were largely related to specific characteristics of the interventions (i.e., strong intervention implementers—facilitator; small number of CHWs involved—barrier). Optimizing the sustainability of breastfeeding interventions in LMICs is imperative to reduce child mortality. The focal point of implementation must be planning for sustainability to lead to continued benefits and changes in population outcomes. A defined action plan for sustainability needs to be included in both funding and research.

## Introduction

In 2020, there were 5.0 million children who died before the age of 5 years (1); that is about 13,698 children who die per day globally. However, the global child mortality rate is the lowest it has ever been at 37 deaths per 1,000 live births down from 93 deaths per 1,000 live births in 1990 (1). Health-sector investments and economic growth contribute to the reduction of child mortality in low- and middle-income countries (LMICs) (2). Even with improved efforts, low- and middle-income countries (LMICs) still average at 41 deaths per 1,000 live births (4.1%) (3), which is a higher than the global average. The range of child deaths is predominately large and burdensome within LMICs, ranging from 2 deaths per 1,000 births (0.2%) in Montenegro to 117 deaths per 1,000 births (11.7%) in both Nigeria and Somalia (3), highlighting the need for implementation and sustainment of interventions to reduce child mortality. Currently, the world is not projected to reach the Sustainable Development Goal (SDG) for child mortality in 2030—to reduce the death of children to a rate of at least 2.5% globally (4), about 25 deaths per 1,000 live births (5). Children under the age of 5 are dying every day from pneumonia and other lower respiratory diseases, preterm births and neonatal disorders, diarrheal diseases, congenital defects, and infectious diseases (4). However, populations and individuals can prevent many under-5 child deaths, yet interventions that save children's lives are not evenly dispersed among children aged from birth to 5 years old. Preventing the death of older children has a predominantly higher percentage of success (65%) compared to that for babies (39%) (4). While older children often die from diseases that can be prevented through vaccinations, babies typically die from pre-and post-term birth difficulties (4). In terms of all-cause child mortality, breastfeeding infants early plays a vital factor in saving their lives (6) because the benefits of breastfeeding are advantageous and extend into adulthood for all children no matter their location.

The World Health Organization (WHO) and United Nations Children's Fund (UNICEF) recommend beginning breastfeeding within 1 h of a child's birth, exclusive breastfeeding (EBF) for at least the first 6 months of the child's life, and introduction of nutritious, complementary foods after 6 months (7). Along with these recommendations, the WHO and UNICEF suggest mothers continue with breastfeeding until the child is at least 2 years old (7). The nutritional content of breast milk changes as a child ages in order to fulfill the child's nutritional needs (8) and allows protection with maternal antibodies to fight infection for both the mother and baby (8). Not participating in or continuing with breastfeeding can increase infant and child mortality (9). Breastfeeding has high coverage rates (10), and LMICs have high percentages of children who are breastfed, but only 37% of children under 6 months are exclusively breastfed (11). In high-income countries, about 1 in 5 children are breastfed for the first 12 months (11).

Many barriers inhibit mothers' ability and desire to breastfeed, such as the marketing of breast milk substitutes industry, access to and education through health care facilities/professionals, lack of resources and/or health insurance, and not an adequate amount of paid maternity leave (9, 12, 13). Though the International Code of Breastmilk Substitutes (“the Code”) was adopted in 1981 to restrict the marketing of breastmilk substitutes, not all countries aligned with the code, and legislation in many countries still has gaps (14). Even in South Africa, an LMIC that is “substantially” aligned with the Code (14), violations of the Code through aggressive marketing tactics have impacted EBF (15). Yet, despite these barriers, if breastfeeding was increased to universal measures, 823,000 children's lives would be saved each year in high mortality rate LMICS (11) because breastfeeding can reduce death due to diarrhea (16), respiratory infections (16), and infectious diseases (17), to name a few (11). In the first 2 years of a child's life, higher risks of child mortality are observed with poor breastfeeding practices, or suboptimal feeding per WHO and UNICEF breastfeeding recommendations (18). The 1-year breastfeeding prevalence is highest worldwide in sub-Saharan Africa (SSA), South Asia, and areas in Latin America (11), yet 1 out of 13 children born in SSA never live to the age of 5 (1, 4). Breastfeeding in these regions is not often sustained until the recommended 2-year mark (11). Scientific literature has been published showing the continued low rates of breastfeeding regardless of the innovative implementation programs, strategies, and evidence (19). Because breastfeeding is a cost-effective intervention to reduce child mortality (19, 20), there is an increasing need to sustain breastfeeding in high mortality areas, LMICs, to uphold the recommended WHO breastfeeding recommendations and contribute to changing the narrative of a child's life.

Sustainability is described in various literature, and according to Proctor et al., an adopted combination of definitions from various scholarly sources, sustainability is “the extent to which a newly implemented treatment is maintained or institutionalized within a service setting's ongoing, stable operations” (21, 22). Shediac-Rizkallah and Bone used three definitions to describe sustainability: (1) preserving advantages brought about through a primary initiative, (2) keeping an existing implemented program and (3) strengthening a community's ability to maintain a lasting intervention after depletion of funds (23). The WHO and UNICEF created the breastfeeding recommendations to encourage mothers to provide their infants and children with optimal feeding for the suggested timeframes. It is well-known that breastfeeding provides children with nutritious benefits that support their overall health and wellbeing (9). Moreover, breastfeeding for longer periods helps reduce rates of infectious diseases (17); children's risk of chronic diseases such as allergies, asthma, diabetes, obesity, irritable bowel syndrome, and Crohn's disease throughout childhood and adulthood (24–32); and the number of under-5 child deaths (7). To aid this, there are a number of global interventions that are designed with a focus on promoting breastfeeding and strengthening breastfeeding behavior to improve child outcomes (20, 33). And while it's evident that not all interventions are successful, the sustainment of breastfeeding interventions is rarely or never considered.

Despite the importance of sustainability, there are several gaps in research. Lack of sustainability definitions or inexplicit explanations of an intervention's continuation is more common than not. Scheirer and Dearing also mentioned the data collection and evaluation process needs to extend beyond program implementation to reach continuance of activities and outcomes (34). Alongside their definition of sustainability as “the continued use of intervention or program components and activities for the maintained achievement of advantageous intervention or program and population outcomes,” the authors presented dependent variables, or sustainability outcomes: (1) continuation of service advantages and outcomes, (2) preservation of original program or intervention activities, (3) maintenance of program created collaborations and partnerships, (4) prolongation of applications and strategies brought about during implementation, (5) preservation of the main issue being addressed throughout the study, and (6) dissemination of intervention and activities to other diverse settings (34). Additionally, they provided what influences sustainability through three independent factors: (1) the intervention's characteristics, (2) components of the organizational or program setting, and (3) components in the environment of the intervention location (34). Though sustainability is not always the end goal, especially if the intervention does not need to be sustained due to undesirable intervention or population outcomes, it should be the key objective if an intervention is needed in a specific area, contingent on research-based evidence (35).

Initiation and duration of breastfeeding are crucial and well-researched, but many systematic reviews fail to explore how to sustain breastfeeding interventions in LMICs or center around implementation or cost-effectiveness of interventions to reduce under-5 mortality. There is considerable research on the implementation of and scaling up breastfeeding practices, but there is limited evidence-based research on if breastfeeding interventions are sustained beyond a certain period; thus, the aim of this systematic review was to determine (i) how breastfeeding interventions are continued or sustained in low- and middle-income countries to reduce child mortality rates, and (ii) identify the barriers and facilitators to the sustainability of breastfeeding interventions in LMICs.

## Methods

### Search strategy

The Preferred Reporting Items for Systematic Reviews and Meta-Analyses (PRISMA) guidelines were used to develop and outline the search strategy (36). We searched Cochrane Library, Global Health, PubMed, Scopus, and Web of Science using the following search terms: (child OR children OR infant OR infants OR neonate OR neonates OR newborn OR newborns OR “under-five child” OR “under-five children”) AND (“child mortality” OR “child death” OR “infant mortality” OR “infant death” OR “neonatal mortality” OR “neonatal death” OR “under-five mortality” OR “under-five death”) AND (breastfeeding OR “breast feeding” OR breast-feeding OR breastfeed OR “breast feed” OR breastfed OR “breast fed” OR “infant feeding” OR “newborn feeding” OR “human milk” OR “breast milk” OR “exclusive breastfeeding” OR “exclusive breast feeding”) AND (“randomized controlled trial”) AND (sustainability OR sustain OR sustainable). We used other systematic reviews relating to breastfeeding implementations and child mortality to help guide our search strategy (18). Language limitations and the setting of LMICs were not applied in the search; countries were assessed manually. The search was from 10/14/20 to 04/07/21.

### Study selection

After identifying articles through the database search, duplicate records were removed, and an initial screening of all titles and abstracts was conducted separately by two authors (AE, CO). The full-text articles with possible significance were also independently assessed by the same authors (AE, CO) using eligibility criteria. We identified relevant articles and performed data extraction for those articles included in this review.

### Definitions

The following table provides a list of evidence-based definitions we used to add credibility and consistency when determining breastfeeding practices and child mortality (Table 1).

**Table 1:** Evidence-based breastfeeding and child mortality definitions.

**Term**	**Definition**
Breastfeeding	Children receive breast milk (including breast milk which has been expressed or from a wet nurse) and are allowed to also receive any food or liquids which includes non-human milks and formulas (37, 38)
Exclusive breastfeeding (EBF)	Infants (<6 months) are fed only breast milk (including breast milk which has been expressed or from a wet nurse) and nothing else, except for oral rehydration salts (ORS), prescribed medicines, vitamins, and minerals (37–39)
Predominant breastfeeding	Infants are predominantly fed breast milk (including breast milk which has been expressed or from a wet nurse) and nothing else, except for certain liquids such as water, water-based beverages, fruit juice, ritual solutions and ORS, prescribed medicines, vitamins, and minerals (37, 38)
Mixed feeding	Infants (<6 months) receive both breast milk and other foods and liquids which includes non-human milks and formulas (39)
Complementary feeding	Children (recommended > 6 months) receive solid, semi-solid, soft foods, or liquids which includes non-human milks and formulas while also breastfeeding (37–39)
Early initiation of breastfeeding	Children who were introduced to the mother's breast within 1 h of birth in the last 24 months (37, 38)
Continued breastfeeding	Children who receive breast milk measured at both ages 12–15 months of age (continued breastfeeding at 1 year) and 20–23 months (continued breastfeeding at 2 years) (37, 38)
Infant	A child who is <1 year old (40)
Child/under-five mortality	The death of a child before the age of 5 years (rate expressed per 1,000 live births) (41)
Infant mortality	The death of a child before the age of 1 year (rate expressed per 1,000 live births) (41)

### Sustainability framework

**Figure 1:**
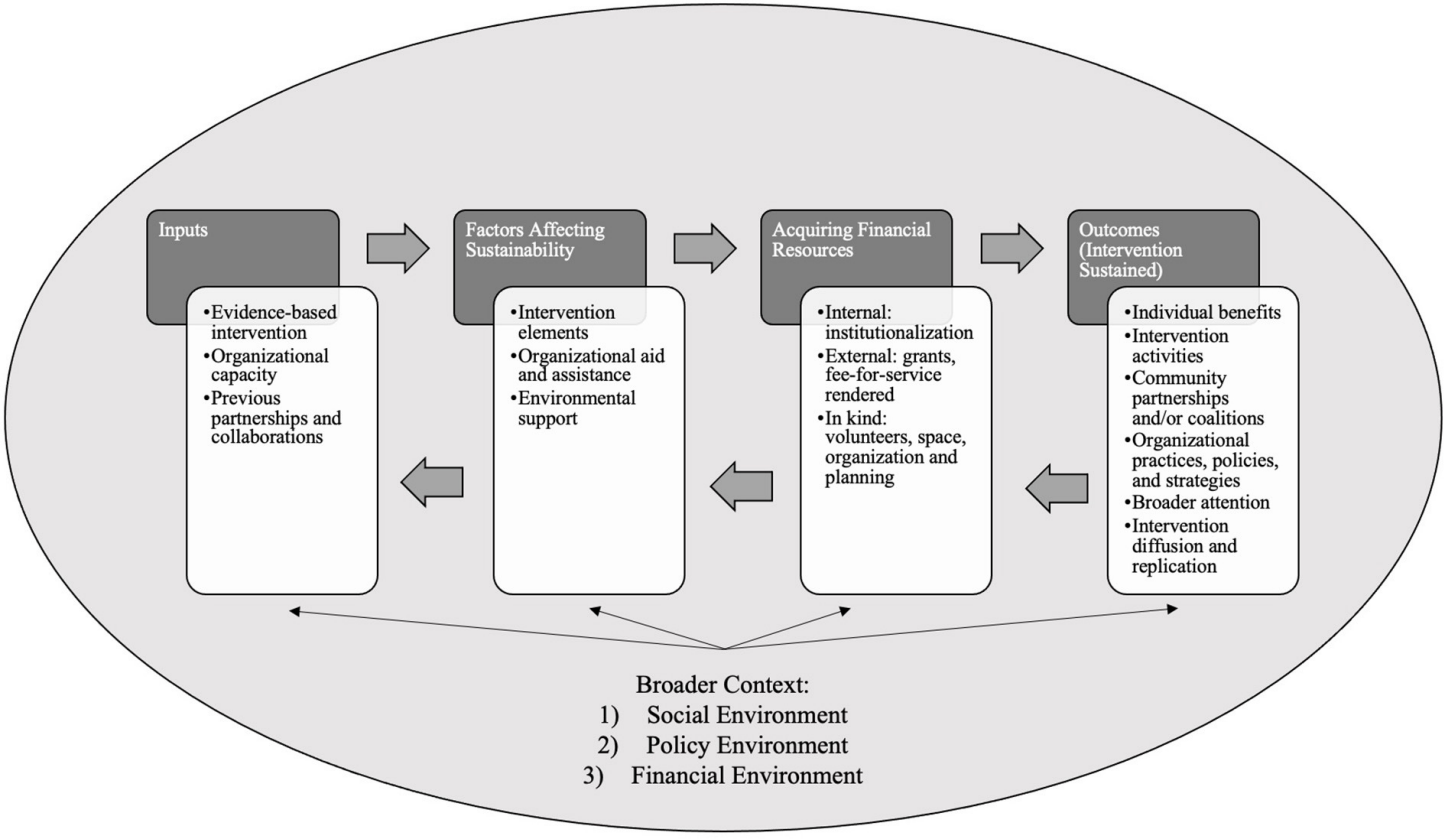
Conceptual sustainability framework.

Sustainability was defined based on the sustainability framework adapted from Scheirer and Dearing (Figure 1) (34). This conceptual framework for sustainability includes factors affecting sustainability (independent variables) and sustainability outcomes (dependent variables) and their placement within the broader context of social, policy, and financial environments (34). This framework displays factors influencing sustainability and outcomes of sustainability are linked with financial resources, and the environments encompassing the organizational environment are impactful to the sustainability of an intervention (34).

### Inclusion and exclusion criteria

Inclusion and exclusion criteria were created, and titles, abstracts, and keywords were examined by two reviewers to determine eligibility for inclusion in the systematic review.

**Figure 2:**
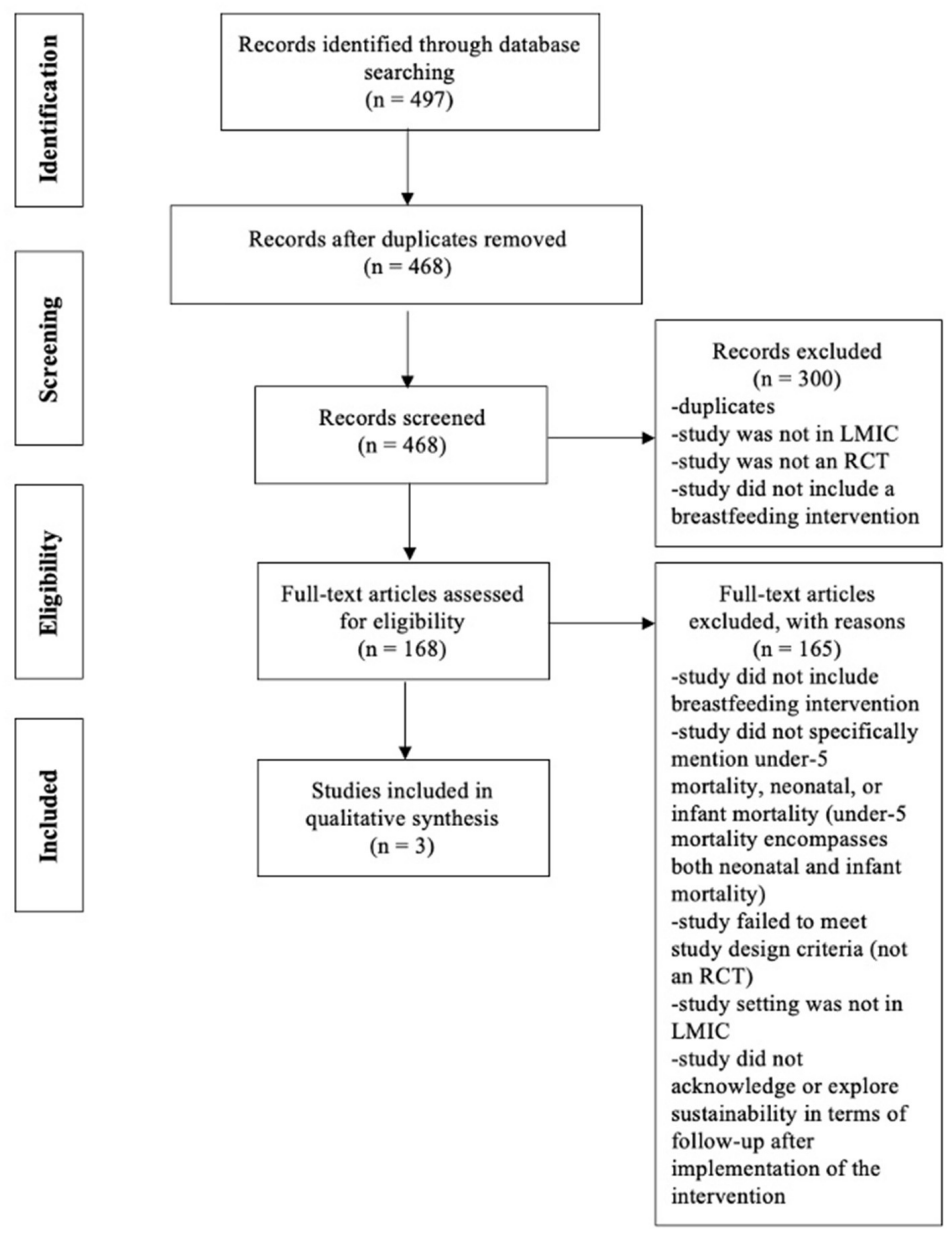
The preferred reporting items for systematic reviews and meta-analyses (PRISMA) flow diagram.

Our inclusion criteria were randomized controlled trials that included (i) infants and children (≤2-years-old) that participated in the initiation of breastfeeding practices, exclusive breastfeeding for the first 6 months of life, or breastfeeding between 6 and 23 months of age, (ii) sustainability of breastfeeding interventions implemented in low- and middle-income countries (inclusion of articles that specifically mentioned breastfeeding and also based on the definition of sustainability provided), (iii) past or current status of breastfeeding practices, and (iv) criteria i-iii related to confirmed or potential contribution to or reduction of child mortality in LMICs. No timeframe was specified for inclusion. The current WHO and UNICEF definitions were used to determine breastfeeding practices (37–39) (Table 1) and child mortality criteria (41), and sustainability criteria were adapted from Iwelunmor et al. (42). Reasons for exclusion throughout the selection of studies, derived from the inclusion criteria, were noted and are summarized in the PRISMA diagram (Figure 2). If insufficient information was included in the paper to determine study eligibility/inclusion in the review, the author of the paper was contacted. If the author did not respond, the study was excluded from the review.

### Data extraction

After assessing full-text articles using predetermined inclusion and exclusion criteria, data extraction was performed separately by two authors (AE, CO). Key concepts and findings from each relevant article were recorded in an excel spreadsheet for comparison. Data extracted included: author and year, intervention country, study population, theory or framework used, outcomes, type of breastfeeding, breastfeeding intervention, the definition of sustainability, and results. A summary table was created to examine the key study details and the sustainability of the breastfeeding intervention included in each study.

### Data analysis

Narrative synthesis, “an approach to the systematic review and synthesis of findings from multiple studies that relies primarily on the use of words and text to summarize and explain the findings of the synthesis” (43), or an analysis of relationships between studies, was used to examine data from the articles in this review. Two authors (AE, CO) independently conducted the narrative synthesis. Narrative synthesis is distinctive for the reason in which it is a literary method to describe study findings (43). There are four main elements of narrative synthesis: (1) development of an intervention theory or framework answering the questions how it works, why, and for whom it is for; (2) development of an initial synthesis; (3) investigation of parallels in data; and (4) assessment of vigor of the synthesis (43). Any discrepancies during screening, data extraction, and data analyzation were discussed until agreed upon by the two authors (AE, CO). If there wasn't agreement, a third author (UN) was brought in to break the tie.

### Risk of bias

The quality and risk of bias of each RCT was assessed using the Cochrane risk of bias tool and reported in Table 2. The Cochrane tool for RCTs assesses five domains: (1) bias emerging from randomization (selection bias,) (2) bias due to veering from planned interventions (performance bias), (3) bias due to absent outcome data (attrition bias), (4) bias in assessing the outcome (detection or measurement bias), and finally, (5) bias in preference of reported result (selective reporting bias) (47). Per the training handbook and tool for randomized trials (47, 48), signaling questions were answered independently to determine the risk of bias for each domain: low risk of bias, some concerns, or high risk of bias. Two authors (AE, CO) assessed the risk of bias for each domain in each article. Discrepancies were noted, and a final decision was determined using a third author (UN), if needed. While each RCT was assessed for quality and risk of bias, no RCT was excluded based on results of the bias assessment.

**Table 2:** Characteristics of included articles in the review.

**References**	**Location/setting**	**Study population**	**Theory/framework**	**Outcomes**	**Mortality**	**Type of breastfeeding**
Daviaud et al. (44)	Umlazi district in KwaZulu-Natal province of South Africa	Pregnant women, ages 17+, and their newborns who were living in the 15 intervention clusters during the recruitment period and provided consent. The study included 30 randomized clusters (15 intervention and 15 control).	None	Primary—assess the effects of CHW antenatal and postnatal home visits through measurements of HIV-free survival, EBF at 12 weeks after birth, care coverage, behavioral indicators (antenatal HIV testing, postnatal visit to clinic within 7 days post birth, uptake of cotrimoxazole for infants subject to HIV exposure, and making use of available family planning practices), and levels of post-partum depression	Neonatal	EBF
Jones et al. (45)	Kiambu County, Kenya	Women from postnatal wards aged between 18 and 40 years old who had a vaginal delivery at 1 of the 3 public health facilities with access to a mobile phone	None	Assess knowledge of danger signs and seeking care related to that knowledge, general postnatal care, and family planning	Maternal and neonatal	EBF
Kumar et al. (46)	Shivgarh, rural block in Uttar Pradesh, India	Pregnant women in 39 village administrative units of 104,123 people total	None	Changes in newborn care applications and neonatal mortality rates	Neonatal	Early initiation of breastfeeding
**References**	**Breastfeeding intervention definition**	**Design description**	**Data collection**	**Definition of sustainability**	**Project timeline**	**Results**
Daviaud et al. (44)	Intervention assessed exclusive and suitable infant feeding at 12 weeks through Community Health Worker antenatal and postnatal home visits	30 randomized clusters of which 15 were intervention and 15 control; CHWs were trained through role plays, demonstrations, real-life experiences, and discussions. CHWs carried out two antenatal visits, a visit within 48 h of birth, four postnatal visits (between days 3–4, days 10–14, 3–4 weeks, and 6–7 weeks), and a final visit between 7 and 8 weeks.	Medical record reviews (routine health data and delivery data) and in-person interview assessments (at 12 weeks postpartum, documentation of CHWs (training, supervising, retention, coverage of visits), 12 week endpoint data and intervention delivery through mobile phones, tool developed by authors to estimate costs, dried blood spots from infants with HIV infected mothers through heel prick (at 12 weeks interview) and tested using DNA PCR testing	Continuation of intervention and scale-up: a multi-purpose CHW now carries out the intervention through Primary Health Care Re-engineering.	Intervention from Jun. 2008–Dec. 2010	EBF prevalence at 12 weeks increased from 15% in the control cluster to 29% in the intervention clusters [Relative Risk 1.92 (95% CI: 1.59–2.33)]. The intervention had a greater effect on mothers who were HIV negative [RR 2.16 (95% CI 1.71–2.73)]. There was not a difference in effect in relation to mothers' education or socioeconomic status. Each additional CHW home visit correlated with a 6% increase in EBF. There was no influence on HIV-free continuation (5.4 vs. 4.5%).
Jones et al. (45)	Breastfeeding included in postpartum checklist messages (“yes/no” questions to assess for insufficient breastfeeding) and general postnatal care messages (general breastfeeding information)	Randomized controlled trial with 4 study arms in which participants were randomized (through a random number generator) into 1 of the 4 groups and uploaded into SMS system: Arm 1-control group in which participants received only standard care (no SMS), Arm 2-intervention group that received postpartum checklist (PPC), Arm 3-intervention group that received PPC plus postnatal care messages (PNC) and reminders 4 weeks post discharge, Arm 4-intervention group that received PPC as well as family planning (FP) messages and reminders 4 and 6 weeks post discharge	Baseline surveys, postpartum checklists “Yes/No” questions throughout intervention, messages tested through focus groups, endline data surveys (8 weeks postpartum)	Continuation and replication of intervention through expansion of access to messaging platform to 5 counties in Kenya, including Kiambu County (setting of study); messaging service now named “PROMPTS”	Enrollment Nov. 2017–Mar. 2018; endline data collection May 2018	Women who received PPC messages were 1.6 times more likely to list 1+ postpartum danger signs (OR = 1.60, 95% CI: 1.07–2.38), 2.57 times more likely to list fever/chills (95% CI: 1.10–5.96), and 3.51 times more likely to seek treatment (95% CI: 1.22–10.07) compared to control group. No difference in general maternal care-seeking or newborn-care seeking behaviors between intervention groups and control. Women who received FP messages were 1.85 times more likely to use FP services (OR 1.85, 95% CI 1.16–2.94), those who were told about FP by healthcare professionals were 2.27 times more likely to use FP services (OR 2.27, 95% CI 1.53–3.35), and women who received FP messages were 2.1 times more likely to use an implant or IUD contraceptive method (OR = 2.10 95% CI 1.06–4.15) compared to controls.
Kumar et al. (46)	Intervention focused on behavior change management aimed toward thermal control and modifying newborn care (birth preparedness, delivery and cod care, thermal care, promoting breastfeeding and recognizing danger signs).	3-arm cluster-randomized controlled trial; control group only received the governmental and non-governmental services, 1 intervention group received the same services as the control group combined with a preventative necessary newborn care package, and the other intervention group was given the newborn care package along with a liquid crystal sticker to identify hypothermia (ThermoSpot). There was 1 community worker per cluster unit. Stratified cluster randomization-39 cluster units were divided among the 3 groups equaling 13 clusters in each group. Volunteers helped with advocacy, building trust, and promoting behavioral changes, and mothers who were great examples of the intervention were used as role models for other pregnant women in the community. Daily and monthly meetings occurred for regional supervisors and their teams. CHWs completed meetings and 2 antenatal and postnatal home visits with intervention groups.	Demographic and socioeconomic indicators collected per household; neonatal deaths and stillbirths assessed through retrospective recall (1 year prior to intervention); knowledge, attitudes, practices, and limitations (maternal and newborn care) collected through a random sample of women who delivered (1 year prior to intervention); pregnancy and birth outcomes identified in study population; baseline surveys identified pregnant women in study areas through 3 monthly door-to-door visits followed with outcome on expected delivery date; 2 door-to-door inspections on pregnancy outcomes; stillbirths and neonatal deaths recorded through questionnaires; knowledge, attitudes, practices, and limitations (maternal and newborn care) collected for those who delivered in study clusters through semi-structured format	Program diffusion, scale-up, and replication: approach is included in the child survival program In Uttar Pradesh and scaled-up through the public health structure.	2003–2006; intervention from Jan. 2004–May 2005	Findings of improvements within the intervention groups were in birth preparedness, hygienic deliveries, newborn thermal care, umbilical cord cutting and care, skin care, and initiation of breastfeeding within 1 h of birth. Adjusted neonatal mortality rate was 54% lower in the newborn care group than control (Rate Ratio 0.46, 95% CI 0.35–0.60, *p* = 0.0001) and 52% lower in the newborn care plus ThermoSpot group than control (RR 0.48, 95% CI 0.35–0.66, *p* = 0.0001).

## Results

### Search results

As documented in the PRISMA diagram, the final database search identified a total of 497 articles. Of these articles and after duplicates were removed, 468 titles and abstracts were screened, and 168 full-text articles were independently assessed using inclusion and exclusion eligibility criteria. Only three randomized controlled trials (44–46) were included in our review after excluding ineligible manuscripts (Figure 2). The characteristics of included articles are shown in Table 2 and described below.

### Characteristics of included studies

Characteristics of the three RCTs that met the eligibility criteria are outlined in Table 2. The studies were published in 2008, 2017, and 2020. The interventions incorporated populations from rural, semi-rural, and peri-urban areas in South Africa (SA), Kenya, and India. Two studies (44, 45) included interventions that evaluated EBF, and one study (46) concentrated on the initiation of breastfeeding. The study populations ranged from 901 (45) to 3,890 (46) participants. Two of the three studies engaged with pregnant women (44, 46), and the other recruited new mothers from postnatal wards (45). The duration of the interventions ranged from 6 weeks (45) to 2.5 years (44). All three studies (44–46) mentioned characteristics of sustainability. However, none used clear definitions of sustainability to describe the continuation of the intervention. Rather, sustainability outcomes of the three RCTs were briefly reported as two of the six dependent variables introduced by Scheirer and Dearing (34).

The included three studies assessed different outcomes using distinct intervention components and data collection methods and measurements. The first study by Daviaud et al. was an economic evaluation of community-based maternal and newborn care from the 2008–2010 South Africa (Goodstart III) cluster-randomized controlled trial (44). This article sought to assess the cost implications of Community Health Worker (CHW) antenatal and postnatal home visits with findings related to the coverage of the intervention, costs of the intervention, and time utilization to determine the sustainability of the program and viability of program replication (44). This paper included background of the RCT, but two other papers, the RCT protocol (49) and a manuscript published on the results (50), were obtained to extract additional data from the study. The intervention was implemented from June 2008 to December 2010 in peri-urban settlement in Umlazi with the costing covering April 2009 to March 2010 (44). The study included 30 randomized clusters of which 15 were in the intervention and 15 were in the control group (44, 49, 50). Participants in the study sample were pregnant women aged 17 and older, who were able to give informed consent to engage in the study, and their newborns in the intervention clusters throughout the recruitment span (44, 49). The intervention's primary outcomes were to gauge the effect of CHW antenatal and postnatal home visits through a set of specific measurements: (1) HIV-free survival, (2) EBF at 12 weeks after the birth of the child, (3) care assurance, (4) behavioral measures (HIV testing before the birth of the child, visit to clinic within 7 days post birth of the child, uptake of cotrimoxazole for babies subject to HIV exposure, and making use of family planning applications), and (5) extent of post-partum depression (44, 49). In terms of breastfeeding, this study's intervention utilized CHWs to assess exclusive and suitable infant feeding at 12 weeks after the child's birth. CHWs were trained thoroughly through a variety of methods such as role-playing, presentations, and conversations (44, 49) to prepare for their antenatal and postnatal home visits to the mothers. CHWs completed eight total visits during the intervention: two antenatal visits; a visit within 48 hours of the child's birth; four postnatal visits between 3–4 days, days 10–14, 3–4 weeks, and 6–7 weeks; and the last visit between 7 and 8 weeks. EBF was recorded for each mother at each visit, and at 12 weeks, mothers participated in in-person interviews with a final assessment of EBF. The intervention proved to be significant in regard to EBF (95%, CI: 1.59–2.33), and a dose-response effect was determined between CHW visits and EBF (6% increase with each visit) (44).

Jones et el. highlighted an RCT study focused on increasing knowledge and pursuit of care behaviors of mothers in peri-urban public facilities in Kiambu County, Kenya, through 6-week short message service (SMS) content intervention (45). Study participants were women aged 18–40 years old from postnatal wards in three public health facilities which assisted individuals from both semi-rural and peri-urban sites (45). Eligible women were those who performed a vaginal delivery at one of the three facilities and obtained a mobile phone (45). Women included in the study were randomized into 1 of 4 study arms and added into a SMS system. The arms were as follows: Arm (1) control group in which participants received only standard care (no SMS), Arm (2) intervention group that received postpartum checklist (PPC), Arm (3) intervention group that received PPC plus postnatal care messages (PNC) and reminders 4 weeks post discharge, and Arm (4) intervention group that received PPC as well as family planning (FP) messages and reminders 4- and 6-weeks post-discharge (45). The primary outcomes of the study were to assess mothers' knowledge of danger signs and seeking care related to that knowledge, postnatal care, and family planning. Outcomes allied to danger signs and seeking care, allied to postnatal care, and allied to family planning were compared to women in the respected arms and then to all women clustered together (45). The intervention gauged EBF using an SMS messaging platform: PPC close-ended, “yes/no,” messages were implemented to evaluate for insufficient breastfeeding and general postnatal care messages including information on breastfeeding, infant care, and family planning were communicated every 3 days after the child's birth from day 6 to 36 (45); FP messages were also included in one arm of the intervention that specifically focused on guidance appertained to 2-year birth spacing, contraception methods, and prompt to remind mothers they can become pregnant after the birth of their child before beginning menstrual periods (45). Significance was identified between participant groups that received PPC messages and those that received FP messages. Participants who received PPC messages were 1.6 times more likely to list postpartum danger signs, 2.57 times more likely to list fever/chills, and 3.51 more times likely to seek further treatment compared to the control group (45). Participating women who received FP messages were 1.85 times more likely to utilize FP services and 2.1 times more likely to employ an implant or intrauterine device (IUD) contraceptive method (45).

Finally, the Kumar et al. study was a cluster-RCT located in a rural area in Uttar Pradesh, India. The trial was a community-based behavior change management intervention that sought to evaluate changes in newborn care applications and neonatal mortality rates (46). Thirty-nine clusters were either randomly assigned to the control group or one of the two intervention groups, equaling 13 clusters per group (46). The control group only received the typical organizational services within the area whereas one intervention group received those same services as the control group with an addition of the preventative necessary newborn care package and the other intervention group was given the newborn care package along with Thermospot (a color changing sticker used to determine hypothermia) (46). The newborn care package included birth readiness, sanitary delivery of the baby, and prompt newborn management: cleansed umbilical cord and skin care, skin-to-skin care, breastfeeding, and seeking care from providers (46). There were 1,141 pregnant women in the control group, 1,600 pregnant women in the first intervention group, and 1,149 pregnant women in the second intervention group (46). To design the intervention, participatory social mapping and qualitative research actions were utilized to learn more about the community and identify and develop an intervention strategy (46). CHWs delivered the newborn care packages to the intervention groups through meetings and four home visits, two before the birth of the baby (60 and 30 days) and two after the birth of the baby (within 24 h of delivery and on day 3 post-delivery) (46). The intervention time span was over 1 year lasting from January 2004 to May 2005 (46). Behavior change management—thermal control and modifying newborn care—was evaluated through door-to-door CHW visits and questionnaires. The findings included improvements in initiation of breastfeeding within 1 h of birth within the intervention groups and in adjusted neonatal mortality rates, with rates 54% lower the newborn care group and 52% lower in the newborn care plus ThermoSpot group than the control (46).

### Narrative synthesis

Facilitators and barriers toward sustaining breastfeeding interventions were identified in the three articles (Table 3). According to Scheirer and Dearing (34), facilitators and barriers of sustainability, or independent variables that affect the sustainability of the intervention, can be categorized into three themes: (1) characteristics of the intervention, (2) factors in the organizational setting, and (3) factors in the community where the intervention is placed, as seen in their conceptual sustainability framework (Figure 1) (34). Facilitators and barriers of the included articles were identified and categorized into the three main categories of sustainment from Scheirer and Dearing (Table 4). Majority of facilitators and barriers were characteristics of the interventions.

**Table 3:** Summary of intervention sustainability.

**References**	**Sustainability outcomes as defined by Scheirer and Dearing: dependent variables of the intervention**	**Facilitators and barriers as defined by Scheirer and Dearing: factors affecting sustainability**	
		**Facilitators**	**Barriers**
Daviaud et al. (44)	Continuation of intervention and scale up: a multi-purpose CHW now carries out the intervention through Primary Health Care Re-engineering	1. Supervision was well-resourced 2. Complex mHealth system was set up 3. Evidence-based intervention effectiveness 4. Multipurpose CHWs during e-engineering of PHC platform	1. High intervention cost 2. Low remuneration of CHWs 3. CHWs spent minimal hours on programme activities (CHW performance) due to several challenges/reasons 4. Concept of “optimal use of CHW time” 5. Small number of CHWs involved 6. Reliability of time monitoring 7. Lack of accountability system for CHWs and supervisors
Jones et al. (45)	Continuation and replication of intervention through expansion of access to messaging platform to 5 other counties in Kenya, including Kiambu County (setting of study); messaging service now named “PROMPTS”	1. Evidence based intervention effectiveness (postpartum and postnatal knowledge and care-seeking behaviors) 2. Family planning messages influenced odds of uptake at 8 weeks postpartum 3. Postpartum checklist supported knowledge and care-seeking	1. Participant resources—reliance on women who own or have access to mobile phones 2. Messaging around postpartum check-ups was broad 3. Generalizability of intervention—phone ownership, literacy, and facility delivery rates—innovation characteristics
Kumar et al. (46)	Program diffusion, scale-up, and replication: the intervention is included in the child survival program in Uttar Pradesh and scaled-up through the public health structure.	1. Evidence based intervention effectiveness 2. Active participation of community members 3. Strong intervention implementers 4. Support from community volunteers and newborn-care stakeholders	1. Behavior change and differing cultural barriers

**Table 4:** Facilitators and barriers as defined by Scheirer and Dearing: factors affecting sustainability.

**Factors affecting sustainability**	**Facilitators**	**Barriers**
Characteristics of the intervention	1. Strong intervention implementers (46) 2. Supervision was well-resourced (44) 3. Multipurpose CHWs during e-engineering of PHC platform (44) 4. Family planning messages influenced odds of uptake at 8 weeks postpartum (45) 5. Postpartum checklist supported knowledge and care-seeking (45) 6. Evidence based intervention effectiveness (44, 46) 7. Evidence based intervention effectiveness (postpartum and postnatal knowledge and care-seeking behaviors) (45)	1. CHWs spent minimal hours on programme activities (CHW performance) due to several challenges/reasons (44) 2. Concept of “optimal use of CHW time” (44) 3. Small number of CHWs involved (44) 4. High intervention cost (44) 5. Low remuneration of CHWs (44) 6. Lack of accountability system for CHWs and supervisors (44) 7. Participant resources—reliance on women who own or have access to mobile phones (resources) (45) 8. Messaging around postpartum check-ups was broad—context (intervention structure) (45) 9. Generalizability of intervention—phone ownership, literacy, and facility delivery rates—innovation characteristics (45)
Factors in the organizational setting	1. Complex mHealth system was set up (44)	None
Factors in the community environment	1. Support from community volunteers and newborn-care stakeholders (46) 2. Active participation of community members (46)	1. Behavior change and differing cultural barriers—context (climate, culture) (46)

#### Characteristics of the intervention, specifically

**Facilitators** Characteristics of the intervention were recognized by all articles as facilitators of sustainability. In South Africa, well-resourced supervision of the CHWs positively affected sustainability of the maternal and newborn care intervention (44). The intervention was noted for its effectiveness and used multipurpose CHWs during re-engineering of the PHC platform (44). A second study by Jones et al. was efficacious particularly with postpartum and postnatal knowledge and care-seeking behaviors (45). Intervention characteristics like family planning messages and postpartum checklists influenced odds of uptake and supported knowledge and care-seeking, respectively (45). In an intervention in India, the implementers of the program, the *Saksham Sahayaks*, played a valuable role in the effect of the study (46).

**Barriers** Several barriers were identified in the articles. Many barriers in Daviaud et al. were related to CHWs such as the limited number of CHWs, the concept of ideal utilization of CHW time and the time CHWs actually spent on program activities, low remuneration of CHWs, and the lack of an accountability for CHWs and supervisors (44). Additionally, the researchers noted that the cost of the intervention was very high (44). Jones et al. recognized that ownership and access to mobile phones, the broad messaging around postpartum check-ups, and the generalizability of the intervention in terms of phone ownership, literacy, and facility delivery rates were barriers to sustain the intervention (45).

#### Factors in the organizational setting, specifically

**Facilitators** Factors in the organizational setting were other facilitators identified. In one article, Daviaud et al., it was found that the complex mHealth system, which was established for research and supervision, aided with data collection, supervision, monitoring, and scheduling (44).

#### Factors in the community environment of each intervention site, specifically

**Facilitators** Factors in the community environment of each intervention site were also categorized as facilitators. Only one article, Kumar et al., communicated facilitators favoring sustainability such as the support from community volunteers and newborn-care stakeholders and active participation of community members for the duration of the research (46).

**Barriers** Only one barrier was identified in the articles. Kumar et al. highlighted behavior change and differing cultural barriers (46).

### Sustainability outcomes

Sustainability of breastfeeding interventions were grouped based on sustainability outcomes (dependent variables), as categorized by Scheirer and Dearing (34) (Table 3).

#### Continuation of intervention

The RCT by Daviaud et al. presented sustainability of the intervention as a continuum of the program through Primary Health Care (PHC) Re-engineering carried out by a multi-purpose CHW, constituting 19% of CHW time for 95% coverage of mothers (44). Leading motives of PHC re-engineering are to improve the geographical context and quality of health, prevention strategies, and health outcomes; enhance efforts of community PHC forces, initiate awareness of social determinants of health, and design a well-structured and effective health system (51). The PHC Re-engineering program goal is to complete seven visits per mother (44).

#### Program dissemination and replication

Kumar et al. mentioned program diffusion and scale-up of the intervention in the study (46). The intervention was accepted as a scale-up framework and approach for expansion and growth and merged into the state's (Uttar Pradesh) public child survival program in India (46). This development and scale-up fosters the newborn care package and extends engagement to over 30 million individuals within the state (46).

The study by Jones et al. yielded both sustainability outcomes mentioned above. This study continued, replicated, and expanded its breastfeeding intervention to five counties in Kenya, including the original setting of the study (45). The findings led to an expansion of opportunity, or increased access, of the messaging platform to women in different counties across Kenya. The study's original enrollment began in November 2017, with endline data collection finalized in May 2018 (45). By May 2020, in just 2 years, and with a new name, PROMPTS, over 150,000 expecting and new mothers enrolled to receive communication from the SMS platform (45).

### Quality of evidence

The articles of RCTs included in the results were assessed for risk of bias (47, 48) and included in Table 5. The risk of bias was similar in all articles, though one of the articles (44) was found to have a high risk of bias arising from the randomization process resulting in a 16.7 % risk of bias overall. The other two interventions had no risk of bias.

**Table 5:** Risk of bias assessed in randomized controlled trails included in review.

**References**	**Bias arising from the randomization process (selection bias)**	**Bias due to deviations from intended interventions (performance bias)**		**Bias due to missing outcome data (attrition bias)**	**Bias in measurement of the outcome (detection/measurement bias)**	**Bias in selection of the reported results (reporting bias)**	**% risk of bias**
		**Effect of assignment to intervention**	**Effect of adhering to intervention**				
Daviaud et al. (44)	High risk	Low risk	Low risk	Low risk	Low risk	Low risk	16.7%
Jones et al. (45)	Low risk	Low risk	Low risk	Low risk	Low risk	Low risk	0.0%
Kumar et al. (46)	Low risk	Low risk	Low risk	Low risk	Low risk	Low risk	0.0%

## Discussion

Several studies have examined breastfeeding through an implementation science lens (52–54), but to the best of our knowledge, there is no article discussing sustainability outcomes in the context of breastfeeding interventions to reduce child mortality. This systematic review aimed to analyze how breastfeeding interventions, with intentions of decreasing child mortality rates, are being sustained in resourced-limited LMICs and identify if any barriers or facilitators that contributed to the sustainability of breastfeeding interventions in LMICs. To our knowledge, this review is the first that looks at the sustainability of breastfeeding interventions in LMICs. It extends the literature on breastfeeding to address child mortality and the area of sustainability in general. Only three breastfeeding interventions in India, Kenya, and South Africa were identified and reported on, and their sustainability was assessed.

Findings communicate that sustainability outcomes of breastfeeding interventions in LMICs were either (1) a continuation of the intervention's activities or components or (2) a diffusion and replication of the intervention as categorized by Scheirer and Dearing (34). Facilitators and barriers toward sustaining breastfeeding interventions in LMICs were largely those of characteristics of the interventions. Facilitators included strong intervention implementers (46), well-resourced supervision (44), use of multipurpose CHWs (44), positive influence of family planning messages (45), supportive postpartum checklist (45), and evidence based intervention effectiveness (44–46). Barriers consisted of a variety of different reasons such as minimal hours being spent on program activities (44), concept of “optimal use of CHW time” (44), not enough CHWs involved (44), high intervention cost (44), low remuneration of CHWs (44), lack of accountability system (44), lack of participant resources (45), broad messaging (45), and generalizability (45). In the organizational setting, specifically, one facilitator was a complex mHealth system (44). In the community environment, there was support from community volunteers and newborn-care stakeholders as well as participation from the community members (46), but a barrier included behavior change and differing cultural context (46). Of the three studies, none included a clear definition of sustainability backed by literature, and there were limited sustainability plans.

With regard to sustainability, the literature reiterates the importance of the timing of research concerning sustainability and the importance of considering sustainability as a set of outcomes or variables rather than a process (34). Not only, it highlights planning for sustainability during the planning and design of the evidence-based intervention rather than after the implementation and evaluation, or latter stages, where researchers generally place the development (55–58). The articles call attention to sustainability as an outcome, but consistent with findings from Iwelunmor et al. (42), planned sustainability efforts were not addressed across all of the studies, even if planned, coinciding with the literature whereby there is, unfortunately, a great deficiency in the planning of sustainability (59). But costing and human resources of the Daviaud et al. Goodstart III intervention were analyzed, and health systems issues of connections to sustainability planning and assurance were identified (44). The study accentuates its goal of developing, assessing, and costing the intervention delivered by CHWs to scale-up and continue the intervention (44, 49, 50). Daviaud et al. highlights the critical call for “planners” to ensure the sustainability of interventions as the lack of financial sustainability of program funding contributes to the collapse of program sustainability (44). Although this is appreciable, the “planners” (44) need to be the researchers. The responsibility of planning to sustain programs is the researchers', the funders', physicians', and program recipients' rather than assuming that sustainability planning is allocated to a “planner” (59).

Not only should sustainability planning be implemented and fulfilled during the intervention development phase, but sustainability definitions and proper use and modeling of evaluated sustainability frameworks should be incorporated within the research (60). This can aide researchers to understand the issues pertaining to precision, adaptation, and essence of the intervention scheme (61). The lack of sustainability frameworks used in research is unfortunately common (60, 61) but framework selection and application should remain a priority.

While it is well-known that there is no universal, confirmed definition of sustainability (62), sustainability was approached in the three studies based on Scheirer and Dearing's sustainability outcomes (34) of continuation of intervention and scale up (44); program diffusion, scale-up, and replication (46); or both (45). Consistent with other literature (63–65), sustainability in these studies reached continuance of activities and outcomes (34). Evidence shows that there is a narrowed focus of sustainability in research, or it is not clearly applied in research (57). In these three studies (44–46), there was minimal reporting on sustainability which made it difficult to determine the extent to which the intervention was sustained. However, these studies identified drivers or barriers that affected the sustainability of breastfeeding interventions.

Earlier reviews have discovered and categorized various facilitators and barriers that affect the sustainability of interventions (55, 61, 63, 66, 67) utilizing different frameworks such as Stirman's influences on sustainability (innovation, organizational context, capacity, and processes) (61), Mays's General Theory of Implementation (capability, capacity, contribution, and potential) (68), Lennox's Consolidated Framework for Sustainability Constructs in Healthcare (initiative design and delivery, negotiating initiative processes, the people involved, resources, organizational setting, and external environment) (69), and Schell's nine domain framework (political support, funding stability, partnerships, organizational capacity, program evaluation, program adaption, communications, public health impacts, and strategic planning) (70). Moreover, few reviews identified facilitators and barriers of breastfeeding interventions (71, 72), but we are unaware of any that particularly assessed those combined, relating to the sustainability of breastfeeding interventions. We categorized facilitators and barriers of interventions in this review by Scheirer and Dearing's factors affecting sustainability (intervention characteristics, organizational setting factors, and community environmental factors) (34), of which other reviews have utilized as well but in different aspects such as youth peer health education network in primary schools (73) and in a school-based bullying prevention program (74). While these factors are similar to those of other frameworks and models, we still see gaps and variations in evidence across the sustainability domain (75).

We are not informed of any other RCTs that specifically define sustainability, plan for sustainability, include a framework of sustainability, and/or discuss the sustainability of a breastfeeding intervention in-depth or even those that explain the reasoning behind an intervention that is not sustained. These gaps present a challenge to evaluate the sustainability of breastfeeding interventions in LMICs. Sustaining a successful intervention should be the objective, but many fall short of this goal in under-resourced locations that need these basic interventions the most. Studies found that were not RCTs primarily accessed breastfeeding knowledge, were not specific interventions for maintaining breastfeeding among mothers, and/or exhibited the relationship between factors and characteristics to lead to breastfeeding practices. Though limited in the number found, the included RCTs provided the information we needed in terms of breastfeeding interventions but occasionally lacked inclusion criteria. Future implications to mind the gap include approaches to address research in practice, specifically, that of sustainability (55). Along with adoption and implementation, sustainability needs to stay at the forefront.

### Limitations

Our review has several limitations. First, a possible limitation would be the specifics of our inclusion criteria that resulted in a limited number of articles in our search. Second, our study is not exhaustive of all studies, but it focuses on evidence-based interventions. Third, the studies did not include definitions of sustainability, nor did they assess sustainability in due course which hosted a challenge when performing a narrative synthesis. Fourth, there is a limited number of peer-reviewed articles pertaining to the sustainability of breastfeeding interventions. This may mean breastfeeding interventions are not sustained so researchers are not documenting the lack of sustainability, researchers are not considering sustainability while planning for and implementing their intervention, or researchers may be avoiding including sustainability in their manuscripts due to lack of knowledge or other specific reasons.

Despite the limitations, there are strengths to this work. According to protocol, this systematic review was completed in a robust manner. A narrative synthesis was used to analyze results, in which a risk of bias assessment (Table 5) was completed for all included studies to establish clarity of our synthesis findings.

## Conclusion

Our findings call attention to sustaining breastfeeding in LMICs to decrease the burden of child mortality. We recommend researchers use implementation science sustainability definitions, frameworks, and literature to guide conceptualization and planning of sustainability of breastfeeding interventions. We also suggest these researchers report on the sustainability of their interventions, whether sustainability was achieved or not (and why), how sustainability was reached, what factors contributed to sustainability, and any challenges faced when managing sustainability. Thorough accountability and communication on sustaining breastfeeding interventions may encourage researchers to follow suit. Future research and interventions should tackle barriers of breastfeeding in LMICs and scale up family- and community-level interventions to foster sustainment of breastfeeding.

## Data availability statement

The original contributions presented in the study are included in the article/supplementary material. Further inquiries can be directed to the corresponding author.

## Author contributions

AE was the first reviewer and main contributor in drafting and writing the manuscript. SM was the second reviewer and contributed to reviewing and editing the manuscript. UN was the third reviewer and contributed to reviewing and editing the manuscript. CO, VC, TS, TG, and DO reviewed and edited the manuscript. The manuscript preparation was supervised by JI. All authors read and approved the final manuscript.

## Conflict of interest

The authors declare that the research was conducted in the absence of any commercial or financial relationships that could be construed as a potential conflict of interest.

## Publisher's note

All claims expressed in this article are solely those of the authors and do not necessarily represent those of their affiliated organizations, or those of the publisher, the editors and the reviewers. Any product that may be evaluated in this article, or claim that may be made by its manufacturer, is not guaranteed or endorsed by the publisher.

## Abbreviations

AE: Alexis Engelhart
CHW: Community Health Worker
CO: Chisom Obiezu-Umeh
DO: David Oladele
EBF: Exclusive breastfeeding
FP: Family planning
IUD: Intrauterine device
JI: Juliet Iwelunmor
LMICs: Low- and middle-income countries
PHC: Primary Health Care
PNC: Postnatal care messages
PPC: Postpartum checklist
PRISMA: Preferred Reporting Items for Systematic Reviews and Meta-Analyses
RCTs: Randomized controlled trials
SA: South Africa
SDG: Sustainable Development Goal
SM: Stacey Mason
SMS: Short message service
SSA: Sub-Saharan Africa
TG: Titilola Gbaja-Biamila
TS: Thembekile Shato
UN: Ucheoma Nwaozuru
UNICEF: United Nations Children's Fund
VC: Victoria Carter
WHO: World Health Organization.
